# Ferroptosis: A Potential Therapeutic Target in Acute Kidney Injury

**DOI:** 10.3390/ijms23126583

**Published:** 2022-06-13

**Authors:** Keiko Hosohata, Tanisorn Harnsirikarn, Susama Chokesuwattanaskul

**Affiliations:** 1 Education and Research Center for Clinical Pharmacy, Osaka Medical and Pharmaceutical University, Osaka 569-1094, Japan; 2 Division of Nephrology, Department of Internal Medicine, Bhumibol Adulyadej Hospital, Royal Thai Air Force, Bangkok 10220, Thailand; t.harnsirikarn@gmail.com; 3 Department of Internal Medicine, Charoenkrung Pracharak Hospital, Bangkok 10120, Thailand; susama.c@chula.ac.th

**Keywords:** ferroptosis, lipid peroxidation, reactive oxygen/nitrogen species, oxidative stress, acute kidney injury

## Abstract

Ferroptosis is a recently recognized form of nonapoptotic cell death that is triggered by reactive oxidative species (ROS) due to iron overload, lipid peroxidation accumulation, or the inhibition of phospholipid hydroperoxidase glutathione peroxidase 4 (GPX4). Recent studies have reported that ferroptosis plays a vital role in the pathophysiological process of multiple systems such as the nervous, renal, and pulmonary systems. In particular, the kidney has higher rates of O_2_ consumption in its mitochondria than other organs; therefore, it is susceptible to imbalances between ROS and antioxidants. In ischemia/reperfusion (I/R) injury, which is damage caused by the restoring blood flow to ischemic tissues, the release of ROS and reactive nitrogen species is accelerated and contributes to subsequent inflammation and cell death, such as ferroptosis, as well as apoptosis and necrosis being induced. At the same time, I/R injury is one of the major causes of acute kidney injury (AKI), causing significant morbidity and mortality. This review highlights the current knowledge on the involvement of ferroptosis in AKI via oxidative stress.

## 1. Acute Kidney Injury (AKI)

Acute kidney injury (AKI) is a life-threatening condition associated with high morbidity and mortality, which occurs in approximately 10–15% of hospitalized patients and in more than 50% of patients in intensive care units [1]. The natural course of AKI varies from complete recovery, partial recovery with stable renal function, becoming chronic kidney disease (CKD), and progressive decline to end-stage renal disease [2]. Severe injury, delayed diagnosis, treatment, and/or repeated AKI episodes increase the risk of potential development to CKD and increase the risk of cardiovascular disease after recovery from the primary insult [3]. Numerous etiologies of AKI have been described. They are sometimes straightforward, for example, renal ischemia due to massive bleeding or profound shock, nephrotoxins, and glomerulonephritis, but, sometimes, they are complicated and multifactorial, such as sepsis-associated AKI and contrast-associated AKI [1].

### 1.1. Ischemia/Reperfusion Injury

Renal ischemia/reperfusion (I/R) injury is triggered by the transient reduction of blood flow to the kidney, followed by blood reperfusion [4]. Both ischemia and reperfusion can induce AKI through several mechanisms, including the generation of reactive oxygen species (ROS), triggering inflammatory cascades, and eventually renal cell death. Tubular cell death of renal I/R injury has the key characteristics of both necrosis and apoptosis, so-called regulated necrosis. Ferroptosis is one prominent form of this regulated necrosis [5]. Renal I/R injury occurs in several situations, resulting in a decrease in circulating blood volume, such as refractory septic shock, massive bleeding, circulatory arrest, or ischemia during kidney transplantation. A meta-analysis showed that the incidence of AKI in patients receiving cardiac surgery was 22.3% [6], which is one of the major causes of renal I/R injury.

### 1.2. Other Kinds of AKI

As mentioned above, there are many causes of AKI other than ischemic insult. Classically, they are classified anatomically into prerenal causes, intrinsic renal causes and postrenal causes. Any pathologies that decrease renal blood flow are categorized as being prerenal causes, including dehydration and non-steroidal anti-inflammatory drug use (NSAIDs). Note that, if the prerenal cause is severe enough, subsequent ischemic injury may also develop. Intrinsic causes are sub-divided into tubular, vascular, interstitial, and glomerular pathologies according to the components of the renal parenchyma. The postrenal causes are those blocking the exit of urine and causing backward pressure on the nephron, and they include bladder stones, benign prostatic hyperplasia (BPH), and bladder tumors [7].

Another common type of AKI is drug-induced. For example, contrast agents induce nephrotoxicity by causing renal vasoconstriction and renal ischemia leading to the generation of ROS and the activation of ferroptosis and cell death [8]. As is the case with contrast agents, cisplatin also causes nephrotoxicity through production of ROS. Thrombin is an important factor in the pathogenesis of cisplatin-induced nephrotoxicity by activation of extracellular signal-regulated kinase (ERK) 1/2, P53, and the caspase-3 pathway [9]. Similarly, cyclosporine is well-known to cause nephrotoxicity [10,11]. Cyclosporine-induced nephrotoxicity is reportedly partly due to renal vasoconstriction [12], and oxidative stress has been reported to be associated with cyclophilin D [13].

Currently, the specific management of AKI involves elimination of the causes, the avoidance and prevention of further insults, and supportive treatment. In patients with ischemic tubular injury, the restoration of circulatory status and elimination of causes are therefore cornerstones of management. Treatment that corrects the cascades following initial ischemic damage to enhance recovery from AKI and decrease the risk of CKD or cardiovascular events is still not available in clinical practice.

Recently, a new type of cell death, ferroptosis, has been discovered [14,15]. Ferroptosis is an iron-dependent regulated cell death, which occurs due to lipid ROS accumulation leading to damage of the plasma membrane by peroxidation of polyunsaturated fatty acids (PUFAs) [16,17]. This type of cell death is involved in the occurrence of degenerative diseases including Alzheimer’s diseases and Parkinson’s diseases [18], stroke [19], intracerebral hemorrhage [20], and AKI [21,22]. The contribution of ferroptosis to AKI via oxidative stress is reviewed here.

## 2. Ferroptosis and Mechanisms

### 2.1. Ferroptosis

Ferroptosis was first defined in 2012, is characterized by non-apoptotic, iron-dependent accumulation of reactive lipid peroxides [14], and its molecular features were recognized as a distinct form. Ferroptosis differs from apoptosis, autophagy, and necroptosis (Table 1), and its morphological characteristics are mainly seen in mitochondria. In ferroptotic cells, shrinking mitochondria are observed, which leads to increased density of the mitochondrial membrane, rupture or vanishing of mitochondrial cristae, and a ruptured outer membrane, whereas the morphology of the nucleus is normal, and the cell membrane remains intact [23,24].

Ferroptosis could be triggered during development or during normal homeostatic tissue turnover, by the accumulation of (1) iron [25,26,27,28,29] or (2) PUFAs [17], or by (3) the depletion of the antioxidant glutathione (GSH) [30], decreased function of glutathione peroxidase 4 (GPX4), which mediates the reduction of lipid peroxides [31,32], or the activation of nicotinamide adenine dinucleotide phosphate (NADPH) oxidase [33,34,35].

### 2.2. Mechanisms of Ferroptosis

#### 2.2.1. Involvement of Iron in Ferroptosis

Iron is an essential element for diverse biological processes, from enzyme activity regulation, immune function, and oxygen transport to mitochondrial function and DNA synthesis and repair. Most iron in humans is used for heme and hemoglobin synthesis [36], acting as the carriers of oxygen to tissues throughout the human body. Two states of iron, the reduced form (Fe(II) or “ferrous”) and the oxidized form (Fe(III) or “ferric”), are present and altered by redox reactions. Iron is therefore an essential cofactor in reactions involving ROS and in handling oxidative stress. Iron is mainly regulated by the liver, reticuloendothelial system, and several mediators. Several organs also have unique local iron regulation [37]. In particular, iron overload is detrimental, affecting several parenchymal organs [38]. Common affected organs in this condition are endocrine glands, heart, and the liver [39]. The kidney can also be damaged by iron. Previous studies found that hemolysis and rhabdomyolysis can cause AKI. They are called hemoprotein-induced kidney injury, pigment-induced kidney injury, hemoglobinuric/myoglobinuric nephropathy, etc. Several mechanisms are responsible for kidney injury in these conditions. Filtered labile iron is one of the contributors to the injury [40]. This reflects systemic iron overload with kidney injury. Recently, an analysis of the ARF Trial Network (ATN) study, which is a multicenter study of critically ill patients, found that elevated plasma labile iron was associated with mortality and AKI requiring kidney replacement therapy (KRT) [41]. This study also highlighted the role of iron in kidney damage in conditions without systemic total iron overload.

Ferroptosis is an iron-dependent form of cell death characterized by the accumulation of lipid peroxidation. Free iron ions accumulate and are catalyzed via the Fenton reaction, leading to the products of lipid peroxides and finally ferroptosis. Similarly, H_2_O_2_ concentration is associated with lipid ROS by the production of HO•. With sufficient GSH concentrations, GPX4 prevents lipid peroxidation that otherwise leads to plasma membrane rupture. FSP1 (also known as AIFM2) prevents lipid peroxidation via a GPX4-independent pathway in the inhibition of ferroptosis. DMT1, divalent metal ion transporter; GPX4, glutathione peroxidase 4; GSH, glutathione; GSSG, glutathione disulfide; H_2_O_2_, hydrogen peroxide; HO•, hydroxy radical; PUFAs, polyunsaturated fatty acids; TFR1, transferrin receptor.

**Figure 1:**
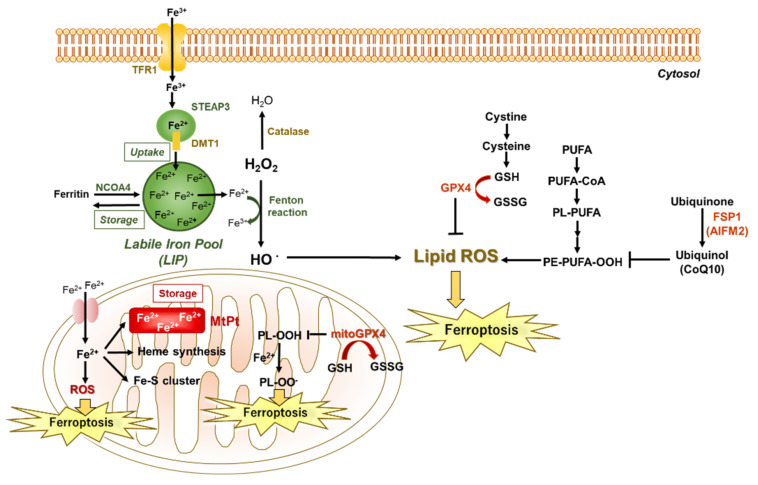
Schematic diagram of ferroptosis.

In physiological conditions, iron entry occurs through the cell membrane by the transferrin receptor (Figure 1). After iron enters the cell, excess iron is usually stored in ferritin or is carried to the mitochondria for heme synthesis and the formation of proteins containing iron–sulfur (Fe-S) clusters [42]. In these clusters, both iron and sulfur can donate or accept electrons and form a crucial component of many enzymes in the body. Abnormal distribution and excess content of iron in cells promotes the Fenton reaction to produce the hydroxyl radical (HO•) and other ROS (Table 2), which can lead to cell and tissue damage [43]. Thus, there is a close relationship between iron overload and ROS, and tissue-iron accumulation can lead to ROS damage and its related toxicities. 

#### 2.2.2. Involvement of PUFAs in Ferroptosis

**Figure 2:**
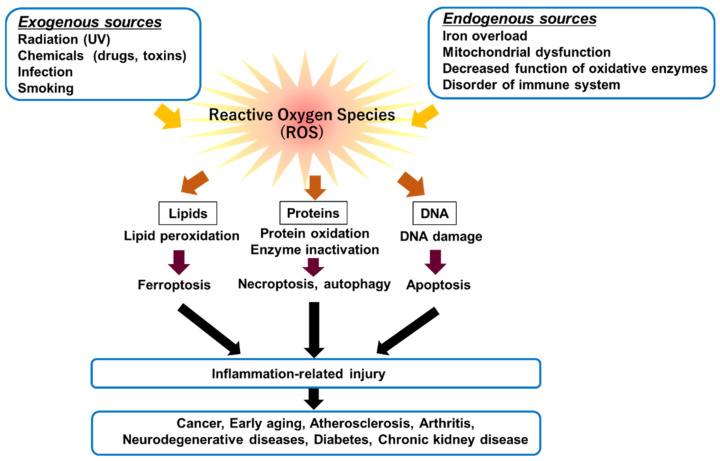
Associations of excessive amounts of ROS with oxidation of biological molecules such as lipids, proteins, and DNA.

The rates of O_2_ consumption in kidney mitochondria are higher than in those of other organs [44], and hydrogen peroxide (H_2_O_2_) release accounts for 0.1–0.2% of the total consumed oxygen [45]. Oxidative stress is an imbalance between ROS production and its removal due to an overproduction of ROS and/or a decrease in antioxidant defense activity [46]. ROS are induced by exogeneous and endogenous sources (Figure 2). The accumulation of cellular ROS can affect cellular contents such as lipids, proteins, and DNA. Major ROS are superoxides (O_2_•^−^), HO•, H_2_O_2_, and singlet oxygens (^1^O_2_), which have highly reactive properties (Table 2). It has been demonstrated that the excessive generation of ROS is harmful to cells, because they cause the oxidation of lipids, proteins, and DNA [47,48]. In particular, O_2_•^−^, HO•, and H_2_O_2_ are detrimental to tissues. Decreased antioxidant capacity and chronic inflammation commonly occur in patients with chronic kidney disease. ROS can react with PUFAs of lipid membranes [16]; PUFAs are subject to lipid peroxidation, and the peroxidated PUFAs drive ferroptosis [17]. Of the PUFA-related phospholipids, phosphatidylethanolamines (PEs) with arachidonoyl (AA), or its derivative adrenaline adrenoyl (AdA), moieties are important substrates of oxidation in ferroptosis [49]. 

The accumulation of cellular ROS can affect or oxidize cellular contents such as lipids, proteins, and DNA. Oxidative cellular damage promotes pro-inflammatory mediator release, which in turn causes ferroptosis, necroptosis, autophagy, and apoptosis.

#### 2.2.3. Involvement of Depletion of Antioxidant GSH, Decreased Function of GPX4, or Activation of NADPH Oxidase in Ferroptosis

The intracellular antioxidant enzyme superoxide dismutase (SOD)1 is the most abundant and can transform O_2_•^−^ to H_2_O [50]. However, the effect of exogenous SOD1 is limited due to its low cell membrane permeability. The further decomposition of H_2_O_2_ to H_2_O and O_2_ is catalyzed by other antioxidative enzymes within mitochondria, such as glutathione peroxidase (GPx) and peroxiredoxin (PRx)/thioredoxin (TRx) [51]. In addition, the depletion of GSH reduces GPX4 activity, leading to the production of excess lipid ROS (Figure 1) [14,23].

Mitochondria are also involved in ferroptosis [52]. Mitochondria are the main organelles of intracellular iron regulation. Cytosolic iron enters mitochondria by mitoferrin1/2 and is used for the synthesis of heme, formation of Fe-S clusters, and storage in mitochondrial ferritin (MtPt). The enhanced accumulation of labile iron in mitochondria induces ROS generation, leading to ferroptosis. Mitochondria have ROS scavenging systems, through which O_2_•^−^ is converted to H_2_O_2_ by SOD, including Cu/Zn-SOD and manganese superoxide dismutase (Mn-SOD) [53]. An excessive accumulation of mitochondrial ROS causes changes in the mitochondrial membrane potential and mitochondrial membrane permeability, leading to mitochondrial damage and cell death [54]. Mitochondrial oxidative stress, associated with the dysfunction of Mn-SOD, is involved in the pathogenesis of various kidney diseases. Mn-SOD function is regulated by post-translational modifications, such as nitration, acetylation, phosphorylation, and glutathionylation. In addition, the mitochondrial respiratory chain (especially complex 1) is a significant superoxide source during renal I/R [55]. During ischemia, when complex I in mitochondria is not oxidizing NADH because of the lack of oxygen, the protein is in an inactive state. Reperfusion of the tissue results in the rapid reactivation of complex I and the generation of large amounts of ROS [56].

There is a close relationship between ROS and reactive nitrogen species (RNS). Major RNS include nitric oxide (•NO), dinitrogen trioxide (N_2_O_3_), peroxynitrite (ONOO^−^), and nitrogen dioxide (•NO_2_), and other oxides of nitrogen. O_2_•^−^ can also react with nitric oxide (NO•) to produce peroxynitrite (ONOO^−^). The most reactive and damaging are HO• and ONOO^−^. These reactants are especially abundantly produced in mitochondria where molecular oxygen (O_2_) is reduced to O_2_•^−^ by electrons that escape from the respiratory chain, especially at mitochondrial complexes I and III. The degradation of ONOO^−^ produces highly oxidizing intermediates, such as nitrogen dioxide (NO_2_•) and the hydroxyl radical (OH•); finally, stable nitrite (NO_3_^−^) is generated. NO synthase (NOS) is expressed at various sites in the kidney [57], with higher •NO levels in the medulla [58]. In general, NO acts as a vasodilator and contributes to lowering vascular tone in the kidney [59]. On the other hand, •NO is produced at the macula densa and is involved in renin secretion and tubuloglomerular feedback via the vasoconstriction of afferent arteries [60].

## 3. The Role of Ferroptosis in AKI

Recently, ferroptosis has been reported to be involved in I/R injury in the kidney [21]. I/R injury in the kidney occurs in two phases [61,62]: the ischemic phase, which is the phase of renal tissue de-oxygenation and ATP depletion, and the re-oxygenation phase that triggers ROS production, inflammatory cascade propagation, and renal tubular damage. Both phases are involved in ferroptosis. In the ischemic phase, the Panx1 channel on the membrane opens and releases ATP, which results in ATP depletion. Extracellular ATP acts as a paracrine molecule and combines with P2Y7 receptors to activate the mitogen-activated protein kinase (MAPK)/ERK pathway, which regulates nuclear receptor coactivator 4 (NCOA4)-mediated ferritinophagy, which induces ferroptosis by degrading ferritin and inducing iron overload [63,64]. In the phase of reperfusion, the rapid restoration of blood flow can induce a burst of ROS production (which is a cause of ferroptosis) and ROS-related injury beyond that of the initial ischemic insult [65]. In addition, renal I/R generates ROS via NADPH oxidase (NOX) [66]. NADP-dependent ROS production is also important for the induction of ferroptotic cell death [14].

Oxidative stress in blood vessels is also involved in renal tissue damage. Chronic hypertension may result from insufficient vasoconstrictor responses. Angiotensin II-induced hypertension promotes an increase in O_2_•^−^ generation through the activation of NOX [53]. ROS derived from NOX are important molecules in endothelial cells and vascular smooth muscle cells, and they are involved in cell growth, migration, inflammation, fibrosis, and contraction [67]. In hypertension, activated NOX (NOX1, NOX2, and NOX4) in blood vessels is related to oxidative stress and abnormal redox signals, leading to the dysfunction of endothelial cells and vascular smooth muscle cells (VSMCs), which causes further vascular damage [68]. Oxidative stress also plays an important role in the progression of vascular calcification [69,70]. Vascular calcification is the deposition of hydroxyapatite crystals in the vasculature [71]. Arterial intimal calcification is related to arterial obstruction and atherosclerotic plaque rupture; on the other hand, arterial medial calcification is related to arterial stiffness, systolic hypertension, and aortic aneurysm [72]. In atherogenesis and atherosclerotic plaque calcification, VSMCs are well recognized as being key contributors. Under healthy conditions, VSMCs are located in the medial layer, where they are responsible for arterial contraction and the production of extracellular matrix, and play important roles in elastic compliance and the recoil of arteries in response to changing hemodynamic conditions. Chondrogenic conversion of VSMCs is increased in atherosclerotic plaques, leading to calcification [73]. Vascular calcification occurs from differentiation or apoptosis of VSMCs and the mineralization of extracellular vesicles in atherosclerotic lesions [74]. Importantly, a contributor to oxidative stress in atherosclerotic lesions is the formation of hydrogen peroxide from diverse sources in vascular cells [75]. Oxidative stress via NOX1 contributes to vascular calcification in patients with CKD, and a NOX1 inhibitor reduces the change [70], suggesting that reductions in oxidative stress via NOX1 may prevent vascular calcification in patients with CKD. NOX5 is a unique homolog of NADPH oxidase since it is Ca^2+^-dependent [76]. Vascular calcification, the formation of calcium phosphate crystals in the vessel wall, results in phenotypic switching from a contractile state to a migrative or proliferative one in VSMCs [77]. Indeed, Ca^2+^-dependent NOX5 increases oxidative stress, leading to an increase in extracellular vesicles, contributing to increased cytosolic Ca^2+^ levels in human VSMCs and subsequent calcification [77].

## 4. Clinical Implications and Future Directions

Removal of the cause of kidney injury is the one of most important aspects of the management of AKI. However, if it is delayed or severe, kidney injury may progress and deteriorate in the absence of a cause through multiple mechanisms. Ferroptosis could be the major pathway in this maladaptive process.

Oxidative stress contributes to drug-induced AKI [78]. These agents and drugs cannot be avoided in clinical practice, since there are no alternative agents. For example, contrast media administration together with computed tomography or coronary intervention is mandatory for the diagnosis of many diseases and treatment of coronary diseases, respectively. The administration of ferroptosis-modulating agents as a prevention may attenuate the chance of AKI development in these at-risk patients.

In the same manner, patients with a risk of developing ischemic renal injury (e.g., patients with massive bleeding with oliguria or elevated biomarkers) may benefit from drugs blocking the ferroptosis pathway. To date, several interventions have been designed to block different nodes of the ferroptosis network, including antioxidants, lipid peroxidation blockade, and iron chelators [79]. Recently, liproxstatin 1, a lipophilic radical-trapping antioxidant, has been used to prevent the propagation of lipid peroxyl radicals and, thus, the peroxidation process. In animal models, the administration of liproxstatin 1 or a ferroptosis inhibitor was shown to reduce iron deposition, cell death, and lipid peroxidation, and to inhibit ferroptosis in renal tubular epithelial cells, eventually inhibiting the morphological changes of renal fibrosis in mice models [80]. Though the interventions are promising for alleviating AKI, clinical studies in humans are still required to confirm their efficacy and effectiveness.

Another interesting example is the use of belatacept, a selective costimulation blocker consisting of soluble CTLA4/IgG fusion protein, to inhibit T cell CD28 signaling, which has been increasingly used to prevent graft rejection after kidney transplantation [81]. Since there is massive I/R injury during kidney transplantation, the possible role of belatacept to prevent I/R injury and the ferroptosis pathway may contribute to higher graft survival. However, further study is needed to completely understand its role.

## 5. Conclusions

Although multiple pathways are involved in the development of AKI, ferroptosis plays an important role in the pathogenesis of AKI because it is involved in the production of ROS. ROS themselves are not harmful; rather, problems arise in relation to the strength and duration of exposure to ROS. Targeting genes involved in ferroptosis and its pathways could provide new therapeutic strategies for AKI, which also needs further in-depth investigation.

In conclusion, research into ferroptosis is still evolving, but it appears to have an important role in the pathogenesis of AKI, and it is one of the promising targets in the treatment of AKI.

## Figures and Tables

**Table 1:** Main features of apoptosis, necrosis, autophagy, and ferroptosis.

	Type of Cell Death			
	Apoptosis	Necroptosis	Autophagy	Ferroptosis
Cell morphology	Shrinkage	Swelling	Accumulation of autophagosomes, double membrane vesicles with multiple cytoplasmic contents	Swollen cytoplasm and organelle, shrunken mitochondria with reduced cristae and ruptured outer membrane
Nucleus	Rupture	Nuclear condensation	Degradation	Normal
Cell membrane	Blebbing	Rupture	Focal plasma membrane rupture	Lack of rupture and blebbing of the plasma membrane
Key protein	Initiation: caspase-2, -8, -9, and -10; execution: caspase-3, -6, and -7	RIP1, RIP3, MLKL	ATG5, ATG7, LC3, p62/SQSTM1	GPX4, GSH
Biochemical characteristics	DNA degradation	Inflammatory response	Increased activity of lysosomes	Lipid peroxidation in cells induced by ferrous or esterase

ATG, autophagy-related; GPX4, glutathione peroxidase 4; GSH, glutathione; MLKL, mixed lineage kinase domain-like; RIP, receptor-interacting protein.

**Table 2:** Classification of reactive oxygen species.

Free Radicals		Non Radicals	
HO•	hydroxy radical	^1^O_2_	singlet oxygen
O_2_•^−^	superoxide anion radical	O_3_	ozone
RO•	alkoxy radical	H_2_O_2_	hydrogen peroxide
ROO•	peroxy radical	ROOH	lipid hydroperoxide
•OOH	hydroperoxyl radical	HOCl	hypochlorous acid
•NO_2_	nitrogen dioxide		
•NO	nitric oxide		

## Data Availability

Not applicable.

## Abbreviations

AKI	acute kidney injury
BPH	benign prostatic hyperplasia
CKD	chronic kidney disease
ERK	extracellular signal–regulated kinase
GSH	glutathione
GSSG	glutathione disulfide
GPX4	glutathione peroxidase 4
H_2_O_2_	hydrogen peroxide
I/R	ischemia/reperfusion
KRT	kidney replacement therapy
NADPH	nicotinamide adenine dinucleotide phosphate
NOX	NADP oxidase
NSAIDs	non-steroidal anti-inflammatory drugs
PUFA	polyunsaturated fatty acid
RNS	reactive nitrogen species
ROS	reactive oxygen species
SOD	superoxide dismutase
VSMCs	vascular smooth muscle cells
